# Novel green flexible rice straw nanofibers/zinc oxide nanoparticles films with electrical properties

**DOI:** 10.1038/s41598-023-28999-x

**Published:** 2023-02-02

**Authors:** Rasha M. Abd El-Wahab, Shaimaa M. Fadel, Amal M. Abdel-karim, Sherif M. Eloui, Mohammad L. Hassan

**Affiliations:** 1 Physical Chemistry Department, National Research Centre, 33 El Bohouth Street, Dokki, Giza, 12622 Egypt; 2 Cellulose and Paper Department, National Research Centre, 33 El Bohouth Street, Dokki, Giza, 12622 Egypt; 3 Advanced Materials and Nanotechnology Group, Centre of Excellence for Advanced Sciences, National Research Centre, 33 El Bohouth Street, Dokki, Giza, 12622 Egypt; 4 Inorganic Chemistry Department, National Research Centre, 33 El Bohouth Street, Dokki, Giza, 12622 Egypt

**Keywords:** Chemistry, Materials science

## Abstract

In the current work, rice straw nanofibers (RSNF) with the width of elementary fibrils (~ 4–5 nm) were isolated from rice straw. The isolated nanofibers were used with zinc oxide nanoparticles (ZnONPs) to prepare flexible nanopaper films. Tensile strength and electrical properties of the prepared RSNF/ZnONPs nanopaper were investigated. The addition of ZnONPs to RSNF nanopaper did not deteriorate its mechanical properties and showed a slight improvement in tensile strength and Young's modulus of about 14% and 10%, respectively, upon the addition of 5% of ZnONPs. Microscopy investigation using scanning electron microscopy (SEM) showed the inclusion of the ZnONPs within the RSNF. Electrical conductivity and dielectric properties as a function of frequency at different temperatures were studied. The ac‐electrical conductivity increased with frequency and fitted with the power law equation. The dc‐ electrical conductivity of the samples verified the Arrhenius equation and the activation energies varied in the range from 0.9 to 0.42 eV. The dielectric constant decreased with increasing frequency and increased with increasing temperature, probably due to the free movement of dipole molecular chains within the RSNF nanopaper. The high values of the dielectric constant and conductivity of the prepared nanopaper films support their use in electronic components.

## Introduction

Cellulose nanofibers (CNF) represent one of the promising eco-friendly bio-based nanomaterials with multi-purpose uses and applications thanks to their biodegradability, good mechanical strength, and high functionality, i.e., presence of hydroxyl functional groups, and the possibility of introducing others by the known chemical routs. CNF isolation from bleached cellulose pulps (without lignin) has been extensively studied from different wood and agricultural residues; the properties of the isolated CNF depend on the lingo-cellulosic source^1^. Recently, the isolation of CNF from unbleached cellulose pulp, i.e., containing lignin, has been studied with the aim to reduce the cost of nanofibers isolation via skipping the cost of bleaching the pulps and also producing nanofibers with different properties due to the presence of lignin with its aromatic structure at the surface of CNF^2^. The presence of lignin, with its relatively less hydrophobic structure than cellulose, onto the surface of CNF can reduce the sensitivity to moisture sorption and thus impart better dimensional stability, as well as better compatibility with hydrophobic additives. CNF could be formed into sheets with high mechanical strength, transparency, and flexibility, known as nanopaper^3^. Nanopaper can hold within its highly nanoporous structure many inorganic nanoparticles with high homogeneity to produce cellulose/inorganic hybrid materials with applications depending on the incorporated inorganic nanoparticles, such as materials for biomedical uses^4,5^, drug release^6^ sensors^7–9^, dielectrics^10–12^, magnetic materials^13^, electrochromic devices, touch sensors, solar cells and transistors^14^. In all these previous studies, CNF used were isolated from bleached pulps from different resources.

One of the great promising aspects that nanoparticles of metal oxides hold in chemical applications is their remarkable ability to chemically absorb a wide variety of molecules, especially organic molecules that are considered as environmental hazards^15^. Among the different metal oxide nanoparticles, ZnONPs are characterized by different interesting physical and chemical properties such as high chemical and mechanical stability, a broad range of radiation absorption, high catalytic activity, electrochemical coupling coefficient, non-toxic nature, a low-cost semiconductor with a broad energy band of 3.37 eV^16^, UV blocking properties, antibacterial activity, and high photocatalytic properties. ZnONPs are eco-friendly and can be used in many areas including optoelectronics and other electronic devices like solar cells, transparent conducting films, chemical sensors, light emitting diodes^17^, pharmaceuticals, cosmetics, and textile industries.

ZnONPs could be prepared using different methods such as chemical vapour deposition^18^ pulsed laser deposition^19^, and sol–gel processing^20^. Among different methods that have been used for preparing nanomaterials is the combustion synthesis (CS), which was chosen to prepare ZnONPs in the current work. Combustion synthesis (CS), or so called self-propagating high-temperature synthesis (SHS), is an effective and low-cost method that saves time, energy, and equipment, for producing fine and nano sized oxide powders compared with the conventional techniques. The formation of high-purity, and many virtual size and shape products, is an additional advantage of the combustion technique^21^. The process uses the heat generated by the chemical reaction between fuel and metal nitrates to convert the metal ions into the target materials; as the heat required to maintain the chemical reaction is supplied from the reaction itself^21,22^.

The flexibility and mechanical characteristics of CNF along with unique properties of ZnONPs have motivated research on producing hybrid nanocomposites from both of them. For example, Ahmadi et al. employed commercial ZnO, CNF, selenium nanoparticles, and gelatine to obtain biodegradable antibacterial/antioxidant films that can be used as packaging material for extending the shelf life of some food products^23^. Lee et al. utilized commercial bacterial cellulose bundles and ZnO to prepare hydrophobically modified ZnO/CNF nanocomposite as a new type of Pickering emulsion system with enhanced UV-blocking and water-resisting performance^24^. Rabani et al. used Core@Shell and RCore@Shell ZnONPs prepared via two-step reaction sol–gel with CNF for producing films with UV protection application^25^. Alhalili et al. synthesized ZnONPs and TiO_2_ nanopowder by co-precipitation and used them with cellulose acetate to synthesize membranes for dithioterethiol removal from water^26^. Aly et al. used ZnONPs, which were synthesized by hydrothermal method, with commercial graphene oxide and cellulose acetate to obtain nanofibers of cellulose acetate containing ZnONPs/graphene oxide for wound healing applications^27^. Rabani et al. employed commercial CNF and ZnONPs that were prepared by precipitation method to obtain ZnONPs-CNF hybrid to be applied in the cosmetic industry^28^. CNF were used with rod-shape ZnONPs to prepare flexible nanopaper for use as humidity and near infrared sensor^29^, a sensor for gases^30^, and electrically conductive films when used with polypyrrole-modified CNF^31^. The CNF used in all aforementioned studies was isolated from bleached cellulose pulp.

To the best of our knowledge, there are no studies on using ZnONPs synthesized by the combustion process, and CNF prepared from rice straw as a composite for electronic applications. Therefore, the aim of the current work was to prepare low-cost nanopaper film with good flexibility and electrical properties by using the unbleached rice straw pulp as a source of the nanofibers with high lignin content and ZnONPs prepared by the economic combustion method.

## Experimental

### Chemicals, reagents and materials

Zinc nitrate hexahydrate and glycine (HAS HMRZEl Laboratories LTD. Netherlands) were used as an oxidizer and a fuel, respectively. Sodium hydroxide, sodium sulfite, sodium carbonate, sulfuric acid, sodium thiosulfate, potassium bromide, potassium bromate, hydrochloric acid, and acetic acid were analytical grade chemicals and used as received from Fisher Scientific (Loughborough, UK).

### Isolation and characterization of rice straw nanofibers (RSNF)

RSNF was isolated from unbleached rice straw pulp as previously described in detail^2^. In the first stage, unbleached rice straw pulp was pulped using sodium sulfite/sodium carbonate mixture (10% or sodium sulfite and 2% sodium carbonate, based on weight of rice straw). The pulp was washed with water till neutrality. The prepared pulp has the following chemical composition as determined by the standard methods of chemical analysis^32^: 14.15% Klason lignin, 54.12% α-cellulose, 14.34% pentosans, and degree of polymerization 903. The total ash content was 16.63% and the HCl-insoluble ash (mostly silica) was 14.89%. In the second stage, nanofibers isolation was carried out using 2% pulp suspension via disintegration using a shear mixer (Silverson L4RT Silverson Machines Ltd. Chesham, UK) followed by fibrillation using a high-shear ultrafine friction grinder (MKCA6-2, Masuko Sangyo, Kawaguchi, Japan). The gap between the disks was − 90 µm and the running time was approximately 140 min.

### Preparation of ZnONPs

ZnONPs were prepared by mixing calculated proportions of zinc nitrate and glycine according to the concepts of propellant chemistry^22^. The mixed precursors were placed in a porcelain crucible in the furnace at 25 °C, and then heated to 400 °C with a heating rate of 10 °C/min for 2 h. At the end of reaction, the crucible with the product was left inside the muffle to cool to 25 °C. The reaction was carried out in an open recipient. After ignition, fragile foam was formed that was easily crumbled into powder.

### Transmission electron microscopy (TEM) investigation

TEM investigation of RSNF and ZnONPs was carried out using a JEOL transmission electron microscope (JEM-2100, JEOL, Tokyo, Japan) with an acceleration voltage of 100 kV. A drop of the suspension was used on a copper grid bearing a carbon film. In the case of CNF, after being air-dried on the copper grid, the sample was stained using phosphotungstic acid solution and left to air dry before investigation.

### Preparation of RSNF/ZnONPs nanopaper films

ZnONPs powder was added to 2 wt.% aqueous RSNF suspension at loadings of 2.5, 5, 10, 15, and 20 wt.% based on the oven-dry weight of RSNF. The mixture was cooled in ice and subjected to ultrasonic treatment using Hielscher ultrasonic processor (Hielscher Ultrasonics GmbH, Germany); 1 cm diameter probe was used and amplitude was set at 75% of the maximum. During the ultrasonic pretreatment, the temperature of the suspension was cooled down with ice to avoid increasing the temperature.

**Figure 1:**
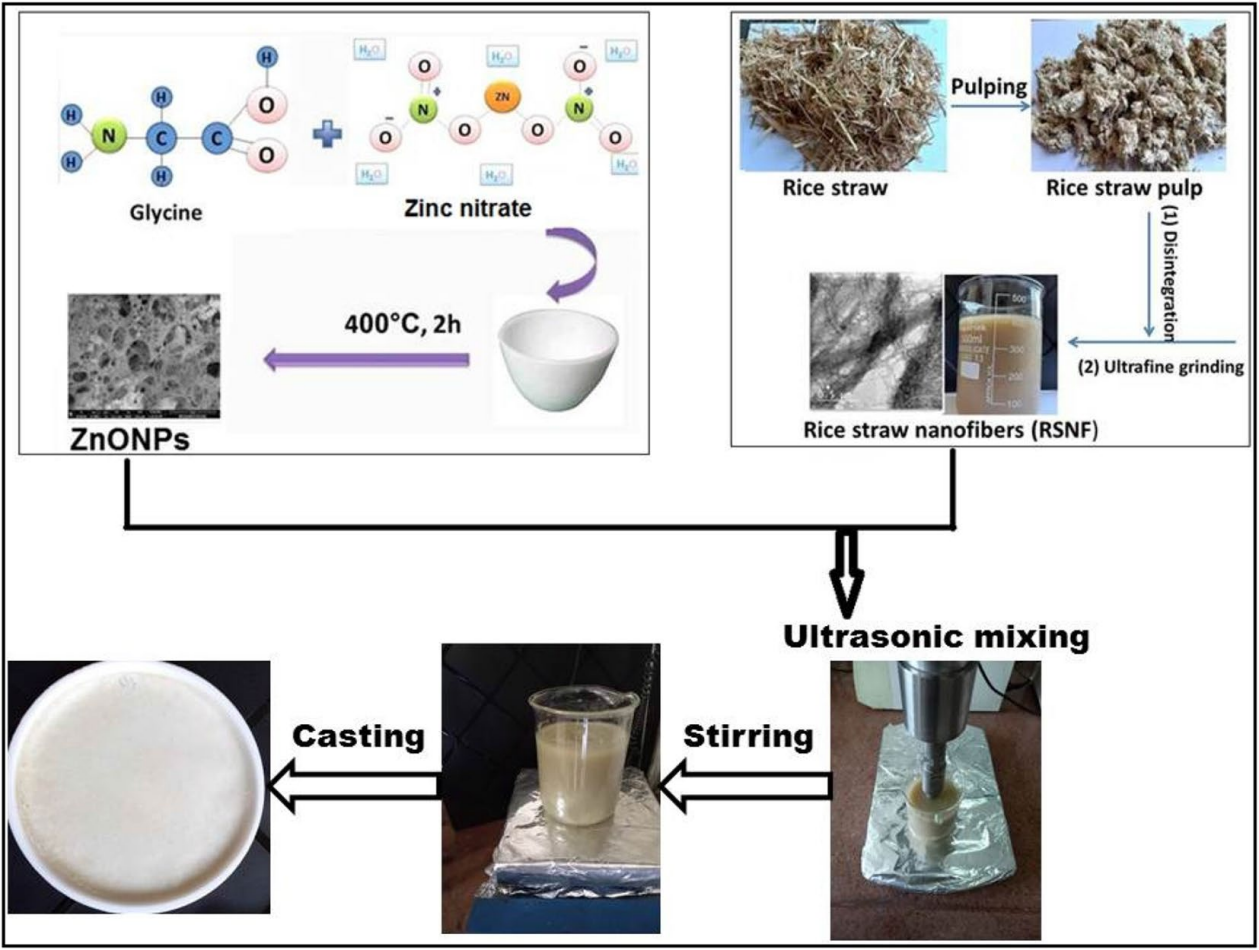
Representative diagram for preparation of RSNF/ZnONPs nanopaper films.

The mixtures were then magnetically stirred at 800 rpm for 4 h to ensure homogeneous distribution of ZnONPs. The films were casted in Teflon plates and dried at 45 °C for 18 h in an oven with circulating air. The scheme of the prepared RSNF/ZnONPs is shown in Fig. 1.

### Characterization of RSNF/ZnONPs nanopaper films

X-ray diffraction patterns were determined using a Bruker diffractometer (Bruker D 8 advance target, Bruker Corporation, Massachusetts, USA ). Cu Kα radiation source with second monochromator (λ = 1.5405 Ǻ) at 40 kV and 40 mA was used and the scanning rate (0.2 min^−1^) was adjusted for phase identification and line broadening profile analysis. The Debye–Scherrer equation (Eq. 1) was used to calculate the crystallite sizes.

1 $$d_{xrd} = \frac{0.89\lambda }{{\beta \cos \theta }}$$

where d_XRD_ is the volume average diameter of the crystallite, λ is the Cu Kα1 wavelength, β is the full width at half of the maximum in radians and θ is the Bragg-angle.

The microstructure of the isolated RSNF was investigated using high-resolution transmission electron microscopy (HR-TEM, JEM-2100 transmission electron microscope, JEOL, Tokyo, Japan). The morphology of the RSNF/ZnONPs samples was determined by high resolution scanning electron microscope SEM Quanta FEG 250 with a field emission gun FEI (FEI Company BV, Netherlands).

The atomic percentages were obtained using Energy dispersive X-ray spectroscopy, EDX, hyphenated with Quanta FEG 250 scanning electron microscopy. The spectra were displayed on TEAM^®^ software at the acceleration voltage 20 kV. X-ray photoelectron spectrometer (XPS, Model: K- ALPHA, Thermo Fisher Scientific, Massachusetts, USA) was operated under the following conditions: (I) Mono-chromatic X-ray Al K-alpha radiation from 10 to 1350 eV. (II) Spot size equals 400 µm. (III) The Applied pressure was 10^−9^ m bar. (IV) Pass energy was 200 eV and 50 eV for a full spectrum and a narrow spectrum, respectively.

### Mechanical properties

Tensile tests of RSNF/ZnONPs nanopaper films were carried out using a Lloyd instrument (Lloyd Instruments, West Sussex, United Kingdom) with a 1 KN load cell. The measurements were carried out on strips with 1-cm width and 9-cm length at a crosshead speed of 2 mm/min at 25 °C. Five replicates of each sample were measured and the results averaged.

### Electrical properties measurement

The electrical conductivity (σ_ac_ and σ_dc_) and dielectric properties ɛ′ and ɛ″ were measured in the temperature range 289 to 373 K and frequency range 100 Hz to 5 MHz using HIOKI 3532 LCR‐Hi‐Tester (HIOKI E. E. Cooperation, Nagano, Japan).

The frequency and temperature dependence of the electrical conductivity were determined from the observed values of the resistance. The conductivity was calculated using the relation

2 $$\sigma = \frac{d}{{R_{p} a}}$$

where *R*_*p*_ is the film resistance, *a* is the cross-sectional area of thin film, and *d* is the film thickness.

The total conductivity is the sum of dc- and ac-electrical conductivity as described in Eq. (3) where dc is frequency independent and ac frequency dependent

3 $$\sigma_{ac} (\omega ) = \sigma_{tot} (\omega ) - \sigma_{dc} (T)$$

where, σ_tot_ is the total conductivity and σ_dc_ and σ_ac_ frequency independent and frequency dependent parts of the conductivity, respectively.

The dependences of the dc-electrical conductivity on the temperature for the studied films were examined and graphically represented. The activation energy E_a_ of the prepared thin film was calculated from the slope of Arrhenius equation:

4 $$\sigma_{dc} \;(T) = \sigma_{0} \exp \left( { - \frac{{\Delta E_{a} }}{KT}} \right)$$

where *σ*_0_ is the pre-exponential factor, T is the temperature in Kelvin and kB is the Boltzmann constant.

Electrical conductivity obeys the Jonscher equation and s values were calculated from the slope

5 $$\sigma_{ac} (\omega ) = A(\omega )^{s}$$

where ω is the angular frequency, A is a constant, and s is the fractional exponent.

The real and imaginary parts of the complex dielectric permittivity ε* (ε* = ε − jε′). The real part of the dielectric function (constant) ε′ of the films was calculated from the measured capacitance at all temperatures and frequencies under consideration according to the equation:

6 $$\varepsilon^{\prime} = \frac{{C_{{\text{p}}} d}}{{a \varepsilon_{{\text{o}}} }}$$

where C is the capacitance in F, *a* is the area in m^2^, and ε_o_ is the permittivity of free space (8.853 × 10^−12^ Fm^−1^).

## Results and discussion

### TEM analysis of RSNF and ZnONPs

**Figure 2:**
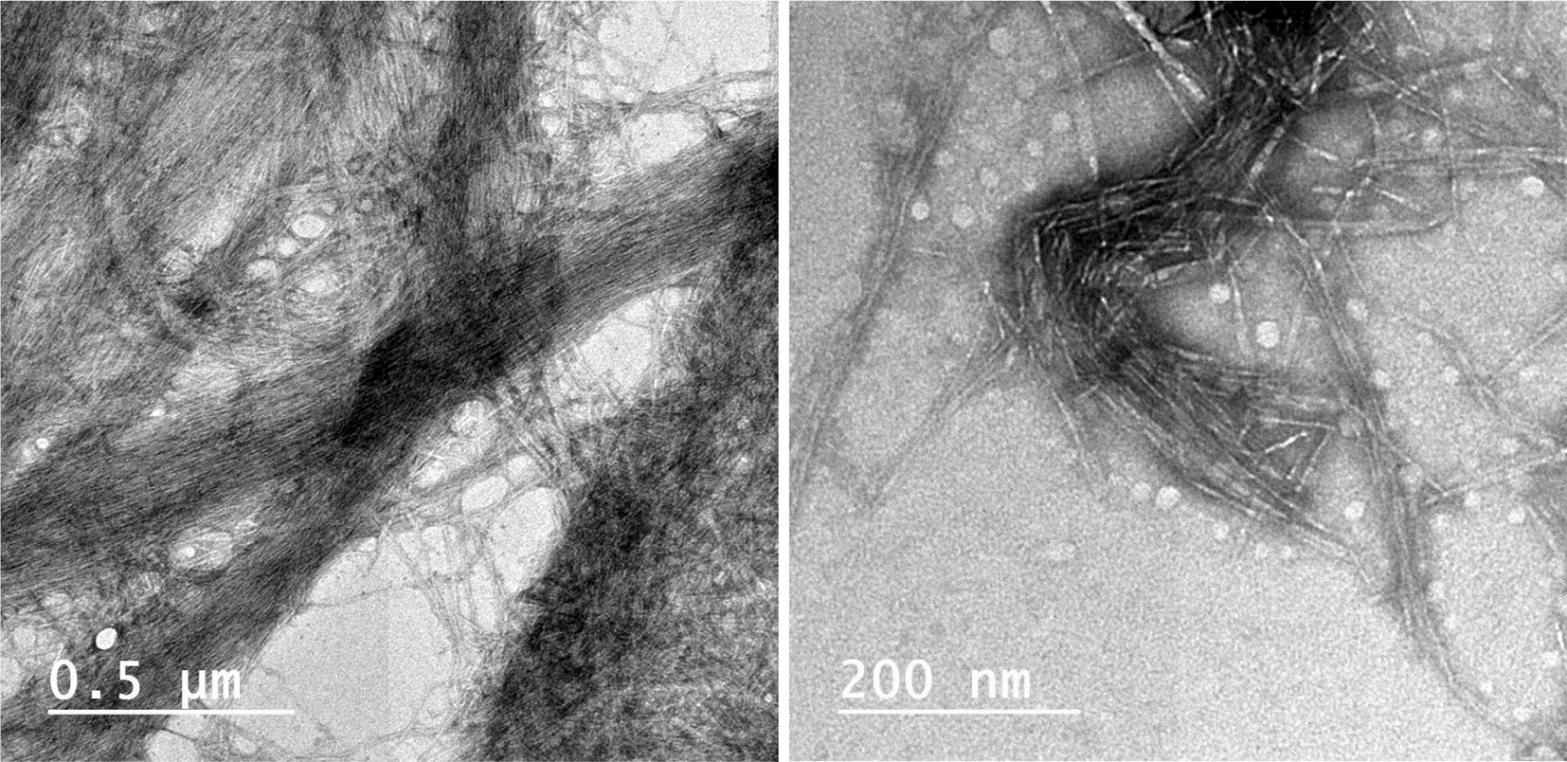
TEM images of rice straw nanofibers (RSNF) at two different magnifications.

RSNF were isolated from unbleached rice straw with about 4 nm width and several microns in length, which is shown in TEM image Fig. 2^2^.

**Figure 3:**
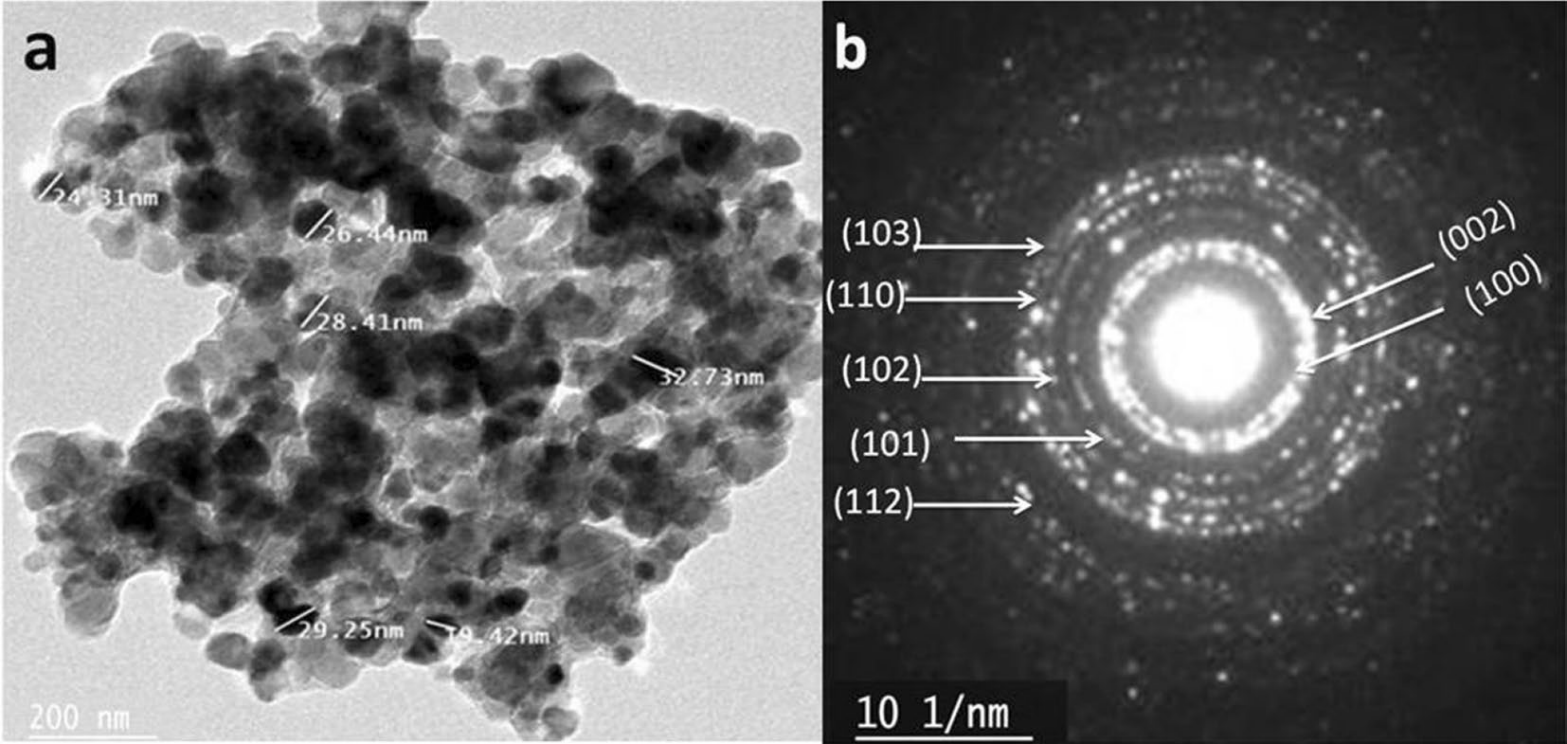
(**a**) TEM image and (**b**) diffraction pattern of as-prepared ZnONPs.

Figure 3a shows the TEM image of the prepared ZnONPs prepared by the combustion method; the average size was 19–33 nm. Polycrystalline structure is obvious from the ring diffraction pattern shown in Fig. 3b**.**

### X-ray diffraction of ZnONPs and RSNF/ZnONPs nanopaper

**Figure 4:**
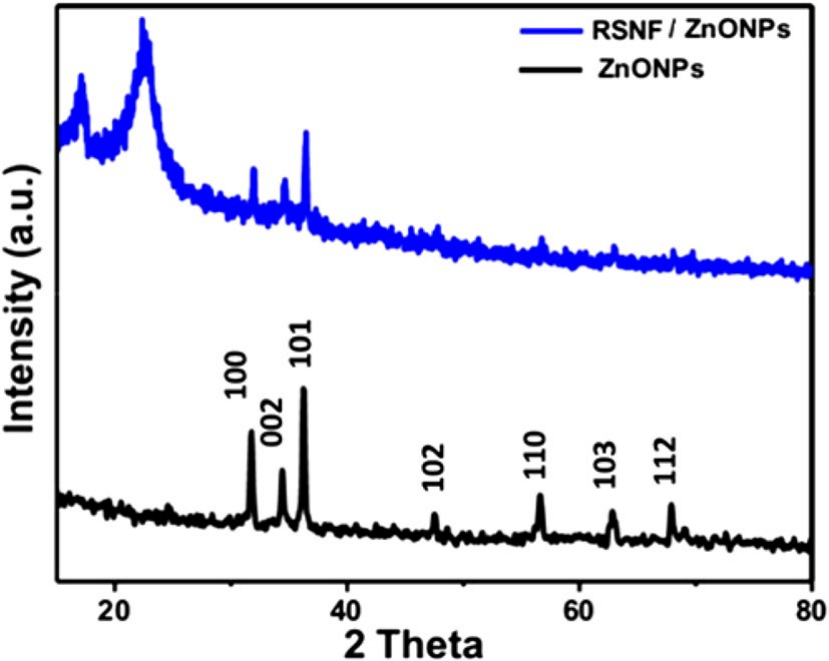
XRD patterns of as-prepared ZnONPs and RSNF/ZnONPs nanopaper film.

The combustion method was successfully employed to prepare ZnONPs. XRD pattern is shown in Fig. 4. According to PDF 05-0664 card, the observed peaks at 2θ = 31.7°, 34.4°, 36.2°, 47.5°, 56.6°, 62.8°, 67.9°, 69.1°,72.6°, 76.9°, 92.9°, and 95.3° are characteristic to the hexagonal ZnONPs with crystal planes (100), (002), (101), (102), (110), (103), (112), (201), (004), (202), (210), and (211), respectively, with a predominant orientation of the crystal plane (101). No peaks related to impurities were detected in the pattern. This confirms that the synthesized powder was single phase ZnO. The crystallite size was calculated for the main peak by the Scherrer equation and was found to be 10.5 nm.

Figure 4 shows the XRD pattern of RSNF nanopaper containing 10% of ZnONPs. The figure clearly shows the diffraction pattern of cellulose I structure with peaks at 22.3, 16.7, and 14.2 due to reflection (200), (110), and (11¯0) planes, respectively^33^. The other peaks from a 2-theta angle from 31° to 70° belong to the crystal structure of ZnONPs as mentioned before. The obtained diffraction pattern means that the addition ZnONPs to RSNF did not affect the crystalline structure of both of them.

### Microscopic structure of RSNF/ZnONPs nanopaper

**Figure 5:**
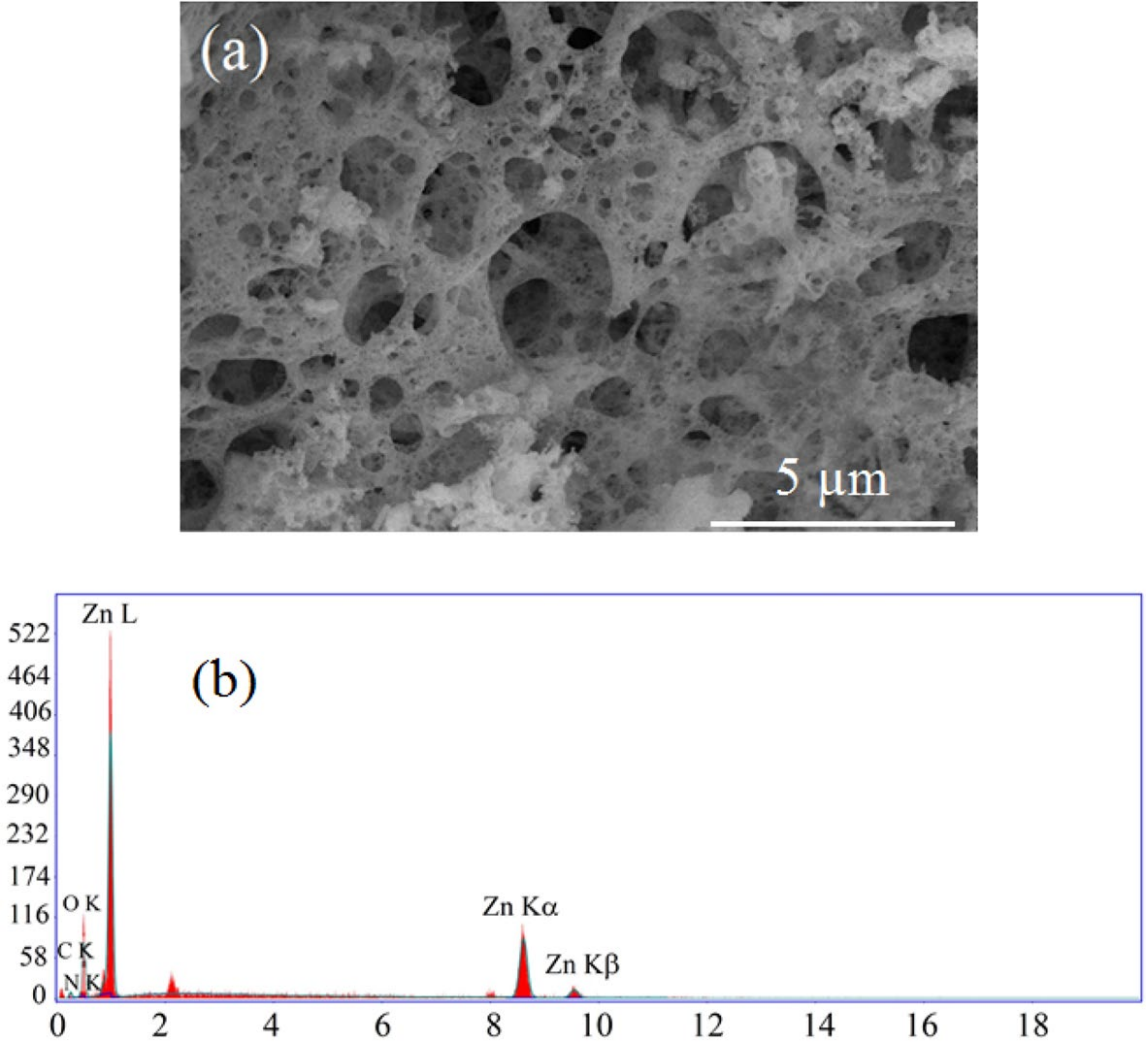
(**a**) SEM image and (**b**) EDX of as prepared ZnONPs, respectively.

Figure 5a shows a friable porous morphology of the prepared ZnONPs. The rapid evolution of the large volumes of the gaseous products during combustion dissipates the heat of the process and limits temperature increase; thus reducing the possibility of premature local partial sintering among the primary particles. The gas evolution also helps in limiting the inter-particle contact resulting in a more easily friable product^21^. Figure 5b displays the EDX spectrum of the synthesized ZnONPs which confirms the existence of Zn and O and demonstrates the successful formation of ZnO. The other minor peaks appeared in the spectrum are due to the gold used in coating the sample before testing.

**Figure 6:**
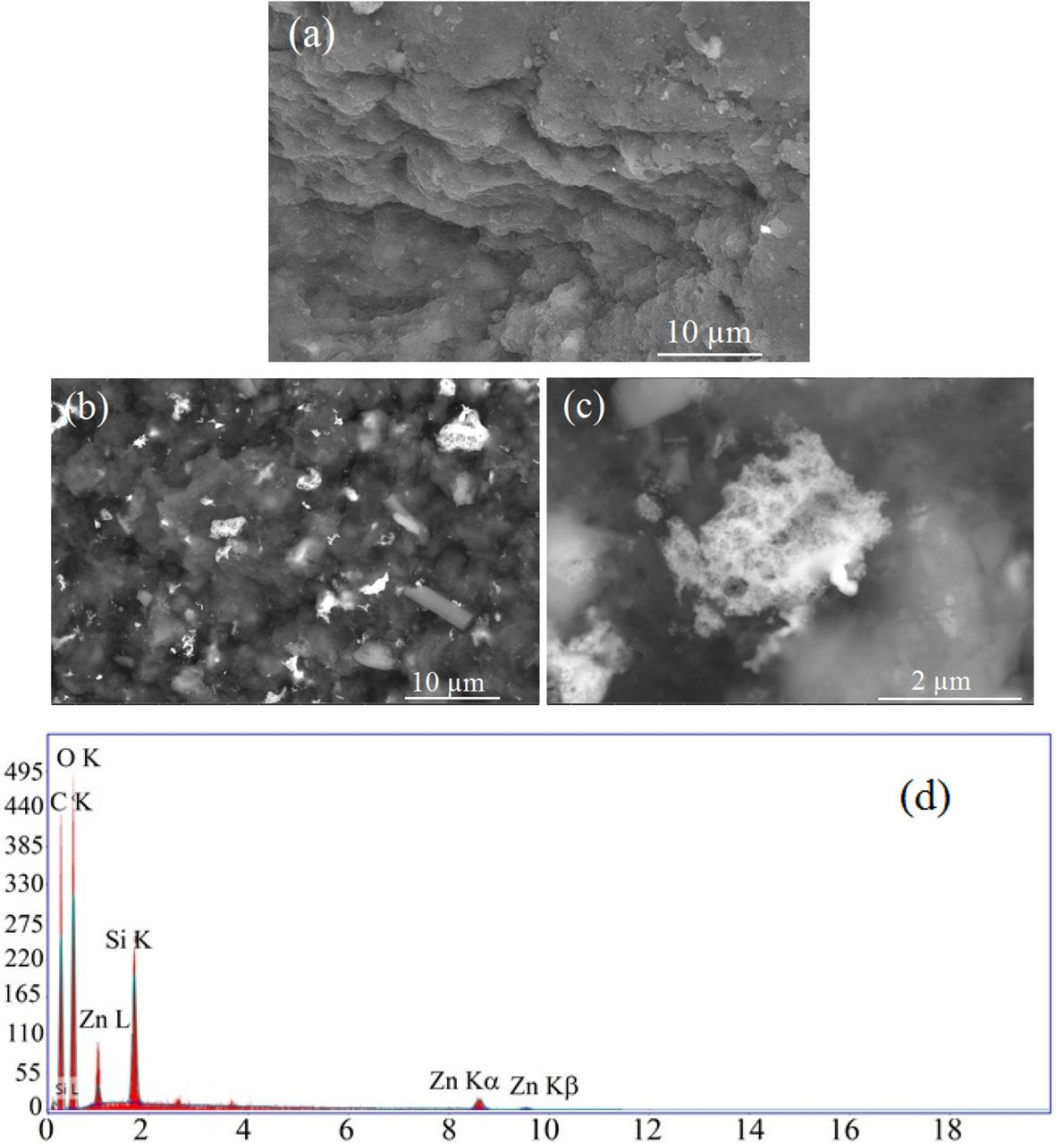
SEM images of (**a**) RSNF nanopaper, (**b**) RSNF/10% ZnONPs nanopaper film at × 8000, (**c**) RSNF/10% ZnONPs nanopaper film at × 60,000, and (**d**) EDX analysis for the surface.

Microscopic structure of RSNF nanopaper and RSNF nanopaper containing 10% of ZnONPs is shown in Fig. 6. The figure shows that RSNF nanopaper has a layered sheets-like structure. On the other hand, the RSNF/ZnONPs film shows ZnONPs embedded into the film and at the surface (images b and c). Due to the very compact and dense structure of nanopaper film obtained upon drying, no separate nanofibers or network of them could be seen at 8000 × or higher magnifications but layers of continuous film only could be seen. High magnification of the ZnONPs at the surface of the film showed its highly porous structure. The EDX analysis of the film's surface (image d) showed clearly the signals of ZnONPs, carbon of cellulose, as well as silica originally found in the rice straw pulp^2^.

### XPS analysis

**Figure 7:**
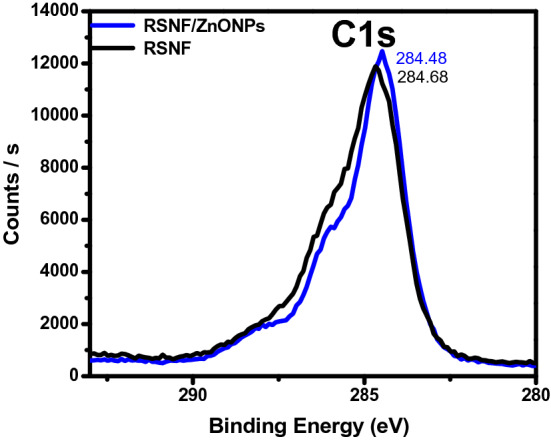
The XPS spectra of C1s of RSNF and RSNF/10% ZnONPs nanopaper film.

The XPS analysis was carried out to investigate the surface chemical composition of RSNF, ZnONPs, and RSNF/10% ZnONPs nanopaper film. Figure 7 shows the XPS decomposition spectra for carbon separately, which are found in the film surface layer that controls the presence of the composition of the rice straw. The figure shows the characteristic peaks at binding energy ~ 285 eV corresponding to the C–C bond. The slight shift of the C (1 s) main peaks from 284.68 in RSNF to 284.48 eV in RSNF/ZnONPs nanopaper film indicates an interaction between RSNF and ZnONPs.

**Figure 8:**
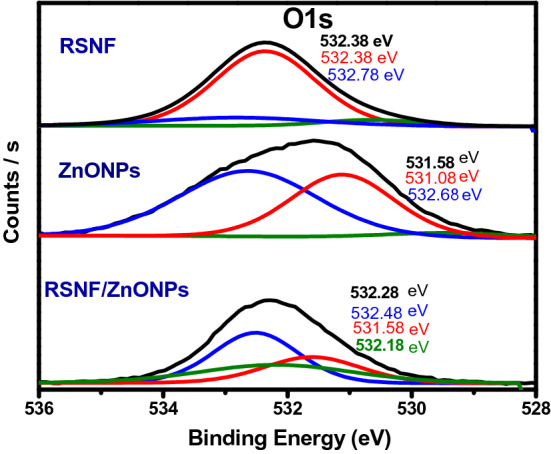
The XPS spectra of O1s of RSNF, ZnONPs, and RSNF/10% ZnONPs nanopaper film.

Figure 8 depicts the O (1 s) spectra of RSNF, ZnONPs, and ZnONPs/RSNF nanopaper. RSNF spectrum showed a peak at 523.38 eV, which corresponds to the oxygenated groups in RSNF^34^. A little peak appeared at 532.78 eV which may be referred to Si–O–Si bonds as the RSNF is rich in silica, as it is previously confirmed by EDX. Hashemi and Bahari^35^ observed Si–O–Si bonds at near value i.e. 532.5. For ZnONPs spectrum, the binding energy of O 1 s is resolved into two peaks at 531.08 and 532.68 eV. The peak at 531.08 eV represents the O 1 s level in the ZnO, which is surrounded by Zn atoms. On the other hand, the peak at 532.68 eV is attributed to the oxygen in absorbed hydroxyl groups. Gogurla et al. showed similar values, i.e., 530 and 531.2 eV, respectively^36,37^. RSNF/ZnONPs spectrum can be fitted into three peaks. The two peaks at 531.58 and 532.48 eV slightly shifted with a significant decrease in the intensity compared to their counterparts in the ZnONPs spectrum i.e., 531.08 and 532.68 eV, respectively. A little new peak appeared at 532.18 eV in the case of RSNF/ZnONPs sample, indicating an interaction between RSNF with ZnONPs nanoparticles.

**Figure 9:**
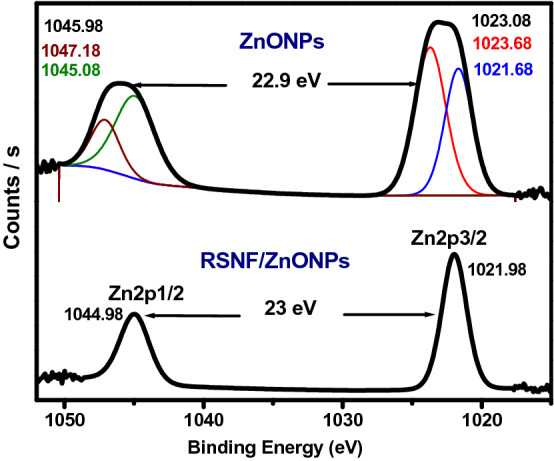
The XPS spectra of ZnONPs and RSNF/10% ZnONPs nanopaper film.

Figure 9 depicts the Zn (2p) orbital binding energy of both ZnONPs and RSNF/ZnONPs nanopaper. ZnONPs spectrum is quite broad, which can be divided into four components located at 1023.68, 1047.18, 1021.68, and 1045.08 eV. The latter two peaks could be ascribed to Zn (2p3/2) and (2p1/2), respectively. However, the appearance of other peaks at about 1023.68 and 1047.18 eV could be attributed to Zn^2+^ in Zn(OH)_2_ as a result of the exposure of the sample to the ambient atmosphere^34^. The components centred at binding energies of 1045.98 and 1023.08 eV are slightly shifted compared to other reports; however, the separation between them is constant and equals 22.9 eV, which is in agreement with previous literature. Gaashani et al. suggest that the binding energies difference could be attributed to two main possible reasons: (1) the chemical environment interaction with the surface atoms. (2) The variation of the texture coefficients with morphology^38^. The binding energy peaks of ZnONPs at 1045.98 and 1023.08 eV shifted to lower binding energy 1044.98 and 1021.98 eV in RSNF/ZnONPs, respectively. The separation between both peaks is equal to 23 eV, which is in agreement with the literature^38,39^. This further corroborates the successful interaction between RSNF and ZnONPs.

### Mechanical properties of RSNF/ZnONPs nanopaper

**Figure 10:**
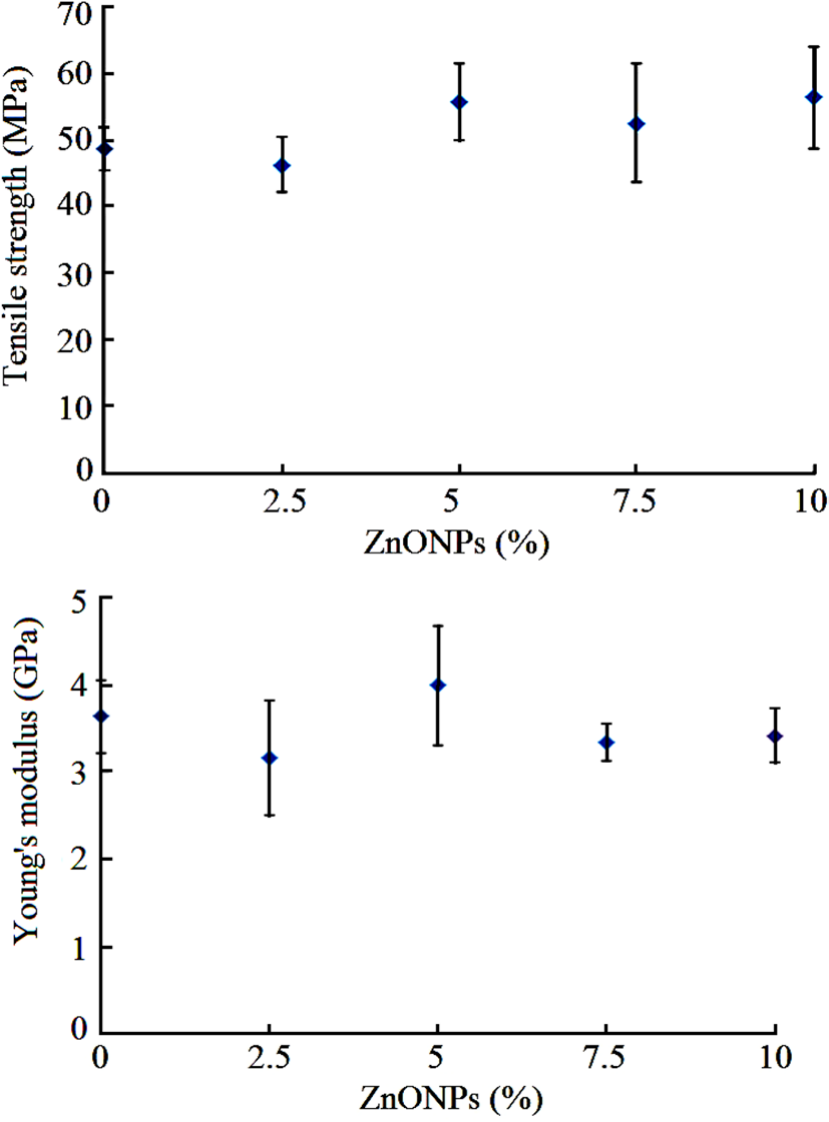
Tensile strength and Young's modulus of RSNF and RSNF/ZnONPs nanopaper films with different ZnONPs loadings.

Figure 10 shows the tensile strength and Young's modulus of the prepared RSNF and RSNF/ZnONPs nanopaper films. The figures imply that addition of the ZnONPs to the RSNF resulted in a slight improvement in tensile strength upon the addition of 5% of ZnONPs; the increase was about 14%. This increase in tensile strength of the RSNF could be due to the high porosity of ZnONPs which may permit the nanofibers to form a network within its porous structure ZnONPs. In addition, the interaction between ZnONPs and CNF proved above could be another reason for the improvement in mechanical properties. However, as the loading of the ZnONPs increases, the hydrogen bonding between the RSNF decreases and thus no further increase in tensile strength was found upon the adding more than 5 wt.% of ZnONPs. On the other hand, the modulus of elasticity of RSNF showed a slight increase (~ 10%) upon addition of 5 wt.% of ZnONPs; at higher loadings, the modulus decreased again but did not go below that of the original RSNF nanopaper film.

### Electrical properties

The electrical properties comprised the dielectric properties, dc- and ac- electrical conductivity.

#### Frequency dependent ac-conductivity studies

**Figure 11:**
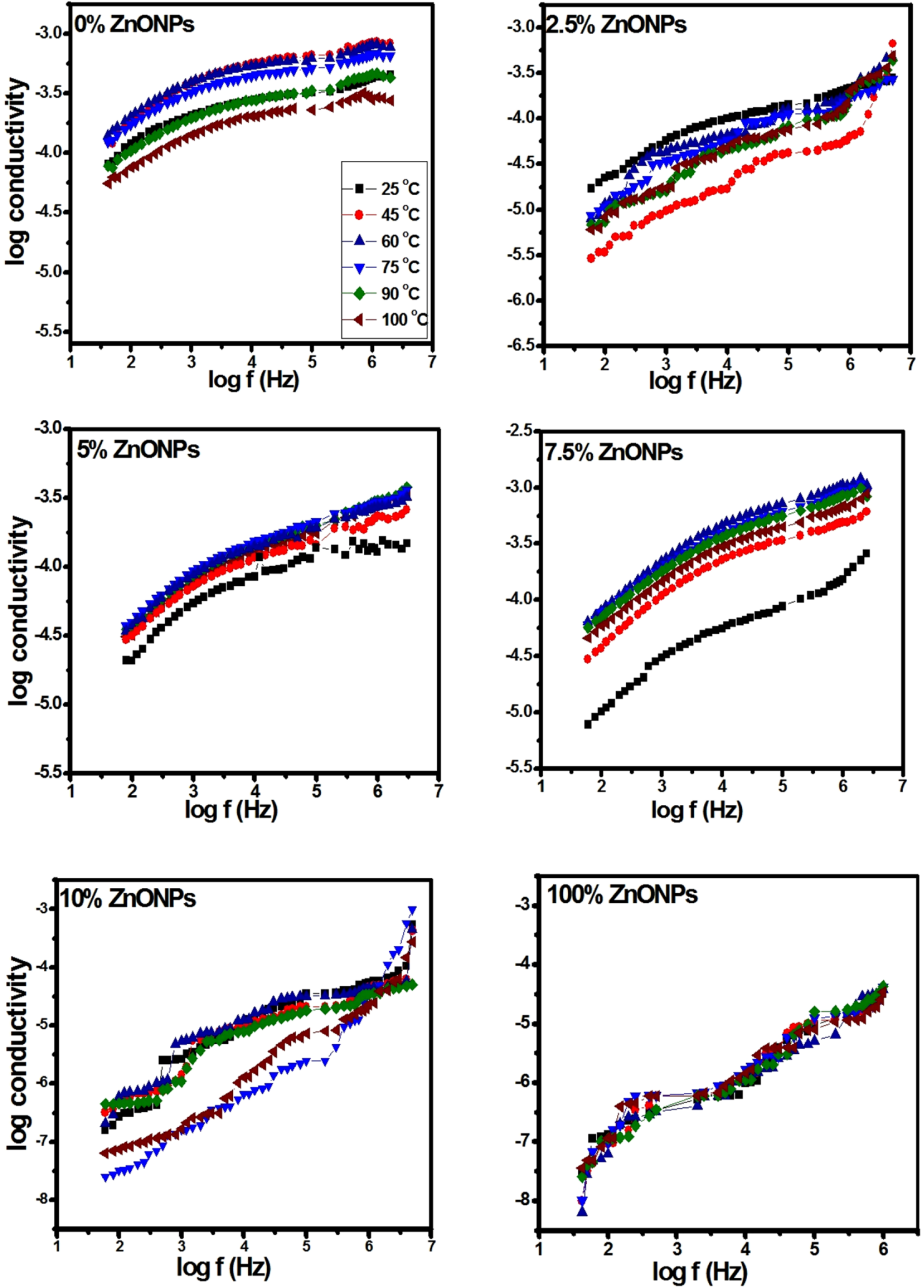
Variation of ac-electrical conductivity with frequency (log scale) at different temperature for RSNF/ZnONPs nanopaper films.

The electrical conductivity σ_ac_ as a function of frequency at different temperatures of RSNF/ZnONPs with different wt.% of ZnONPs is represented in Fig. 11. In all curves, three regions are found: region I in the low frequency where the electrical conductivity resulted from dipole polarization, region II intermediate region, where the dipolar relaxation occurred depending on the wt.% ZnONPs, and region III at the high frequency where the conductivity increased at all temperature ranges due to movements of free charges and bound charges. The cellulose chain has faster internal modes as a result of the increase of the amorphous region where bond rotations produce segmental motion^40^. The conductivity of cellulose is ionic, affected by a number of mobile charge species and segmental mobility of the chains. For the neat RSNF (0% ZnONPs), the plotted curves show clearer balance and tend to present increasing of conductivity with frequency but it decreased with increasing temperature. However, in the case of 7.5 wt.% ZnONPs, the conductivity increased with increasing temperature showing a behaviour similar to that of a semiconductor. The ac-electrical conductivity of neat RSNF increased systematically with increasing frequency due to the increase in the number of charge carriers resulting from the presence of the OH groups in such films.

At room temperature, the conductivity value of RSNF ranged from 8.08 × 10^−5^ at low frequency to 5.99 × 10^−4^ at high frequency, lowest concentration at 2.5 wt.% ranged from 1.15 × 10^−5^ to 2.15 × 10^−4^, 5 wt.% from 1.08 × 10^−5^ to 3.6 × 10^−4^, 7.5 wt.% the value increased from 6.34 × 10^−6^ to 1.22 × 10^−4^, and for 10 wt.% the value increased from 3.58 × 10^−5^ to 5.35 × 10^−4^, which indicates the semiconductor properties for all concentration. The increase of conductivity to 1.22 × 10^−4^ with 7.5 wt.% ZnONPs introduced in RSNF is due to the ions migration between coordinating sites^41^ and improvement of ionic conductivity with the addition of ZnONPs; then decreases with the increase ZnONPs to 10 wt.%.

In addition, the interaction of ZnONPs with RSNF reduces the intermolecular interaction of the RSNF chains; hence the increase of segmental mobility led to greater disorder in RSNF^42^. The relatively amorphous nature of cellulose allows easy migration of ions between ZnONPs and the RSNF matrix^43^. A fine dispersion of ZnONPs at low wt.% increases the interaction with the RSNF matrix enhancing the conducting pathways, thus increasing sites for ion hopping leading to increased conductivity^44,45^. Adding 10 wt.% ZnONPs lead to a decrease in conductivity; this may be due to the association of ions causing aggregation^43^.

All films have semiconductor properties and satisfy the universal power law shown in Eq. (5). Where the *s* value represents the degree of interaction between the mobile ions and the surrounding environment^46^.

It is calculated from the slope of the logarithmic plot of Fig. 11. A smaller value of *s* for the RSNF film was found, i.e., low interactions lead to higher mobility of charge carriers. A film having a higher s value, i.e., higher interaction implies lower mobility, which justifies the fact that sample 10% is the least conductive film. It is the temperature and frequency dependence that produce the nature of the conduction mechanism which may take place in the films^47^. For the Correlated Barrier Hopping mechanism (CBH) *s* values decrease with temperature^48,49^. The values of *s* (*T*) for an ideal Debye dielectric dipolar-type and ideal ionic-type crystal are 1 and 0, respectively^46^.

These calculated values of *s* at the temperature range of 25–100 °C are listed in Table 1. It is observed that the *s* values ranged from 0.8 to 0.16 (less than 1). The *s* values decreased with the increase of temperature for 5.0 and 7.5 wt.% ZnONPs; This supports the assumption that a conduction mechanism of hopping of the charge carries in localized states of RSNF, with the excitation of carriers to upper states in the conduction band^50,51^.

**Table 1:** Variation of s values with temperature.

ZnONPs Wt.%	Temperature, °C		
	25	60	100
2.5	0.17	0.21	0.26
5	0.18	0.16	0.16
7.5	0.23	0.22	0.22
10	0.50	0.38	0.80

#### Temperature dependent conductivity studies

**Figure 12:**
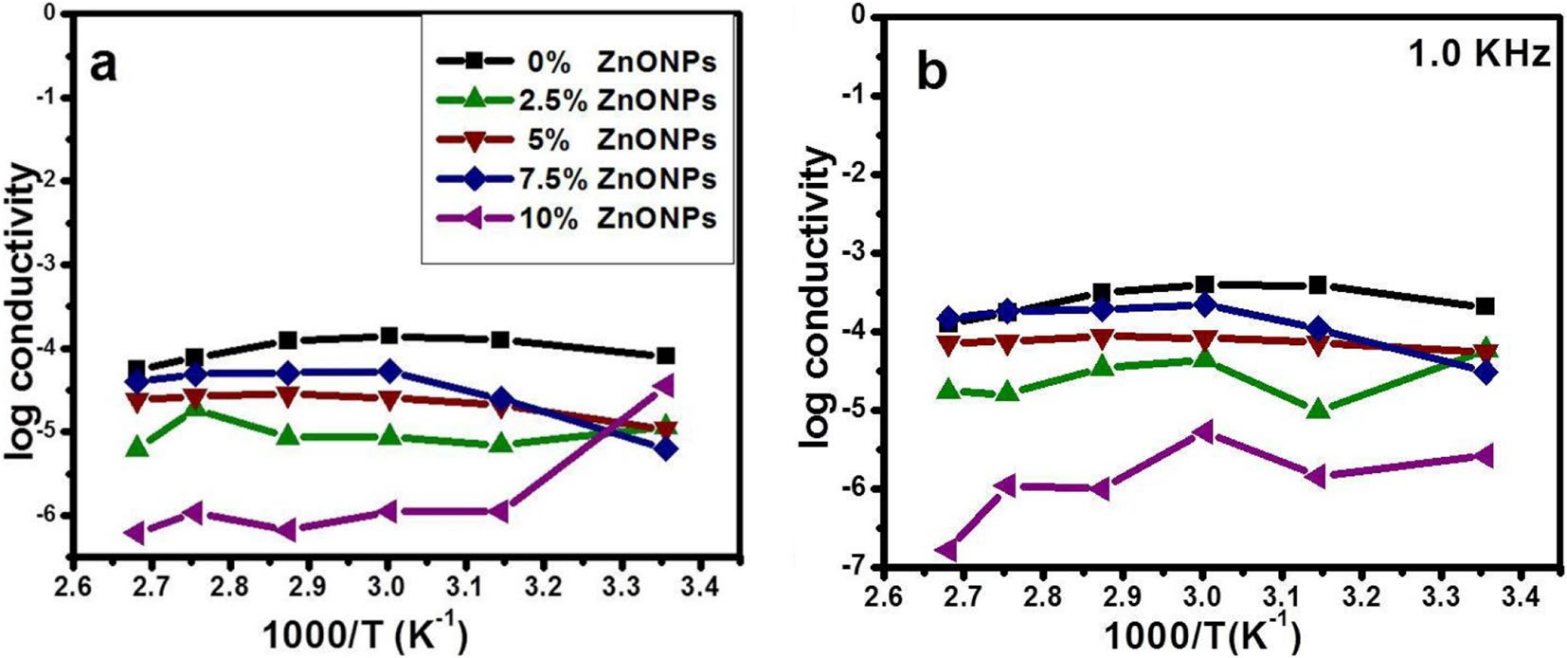
Variation of conductivity of RSNF/ZnONPs nanopaper films with temperature: (**a**) dc-electrical conductivity and (**b**) ac-electrical conductivity.

Figure 12 shows the variation of conductivity with temperature (*σ*_*dc*_ vs 10^3^/T) (*σ*_*ac*_ vs 10^3^/T) at fixed frequency during measurements. All these plots are linear confirming their Arrhenius behaviour over the investigated temperature range from 25 to 100 °C. A trend of the change in the conductivity behaviour is strongly dependent on the concentration of ZnONPs added; ZnONPs were less conductive than that of neat RSNF. With the increasing temperature, the curves showed small fluctuations. Only the sample with 10 wt.% ZnONPs manifested stronger fluctuations in conductivity tending to a gradual decrease with the rise in temperature^52^. The activation energy could be estimated for the set of samples and was calculated based on the Arrhenius equation (Eq. 2), and was represented in Table 2.

**Table 2:** The value of electrical conductivity and the activation energy.

Wt.% ZnONPs	σ_dc_		*E*_a_ (ω)	E_a_ (A)	σ_ac_	
	25 °C	100 °C			1 kHz	1 MHz
0	8.08 × 10^−05^	5.56 × 10^−05^	0.231	0.3	2.07 × 10^−04^	4.38 × 10^−04^
2.5	1.15 × 10^−05^	6.31 × 10^−06^	0.283	0.83	6.21 × 10^−05^	1.47 × 10^−04^
5	1.08 × 10^−05^	2.41 × 10^−05^	0.350	0.22	5.44 × 10^−05^	1.38 × 10^−04^
7.5	6.34 × 10^−06^	4.0 × 10^−05^	0.500	0.50	3.07 × 10^−05^	8.79 × 10^−05^
10	3.58 × 10^−05^	6.20 × 10^−07^	0.424	0.59	4.38 × 10^−07^	2.62 × 10^−06^

The values of ∆E_a_ less than 0.2 eV are generally associated with an electron mechanism of conduction; while values more than 0.6–0.8 eV can be considered ionic transport^53^. The low activation energy for the neat RSNF film can be related to the reinforcing effect of cellulose nanofibers. E_a_ can be given as the sum of strain energy (E_a_) and binding energy (E_b_)^54^. E_b_ is the energy required for an ion to leave its site, while E_s_ is the kinetic energy of the ion that needs to distort its structural environment so as to form a “doorway” through which it can jump to another site. As a consequence of the increased E_s_ due to the heterogeneity of cellulose nanofibers, E_a_ the total activation energy increases. From the values listed in Table 2, it can be inferred that the conduction mechanism for the dc-electrical conductivity σ_dc_ is of an electronic nature. The low value of activation energy also implies that a hopping conduction mechanism is the dominant one for dc- electrical conductivity. They produced value verifying Arrhenius equation activation energy in the range 0.9–0.42 eV. It is clear from the table that the values of *E*_a_ decrease with an increase in wt.% ZnONPs doped in RSNF suggest that the increasing charge carriers are responsible for conductivity. Thus, an increase in temperature produces more free volumes, which increases the mobility of ions and hence conductivity^55^.

#### Effect of ZnONPs concentration

In neat RSNF film, the cellulose polymer formed by d-glucose units connected through (1,4) glycosidic bonds^56^ has a structure forming linear chains with a highly polar surface emerging from multiple hydroxyl groups^57^. Thus, cellulose nanofibers are being vastly used as a matrix to provide electrically conducting, semiconducting or insulating materials^52,58^. The properties of cellulose nanofibers depend on their sources, extraction, and purification procedures factors that should be kept uniform while studying the effect of any additions.

**Figure 13:**
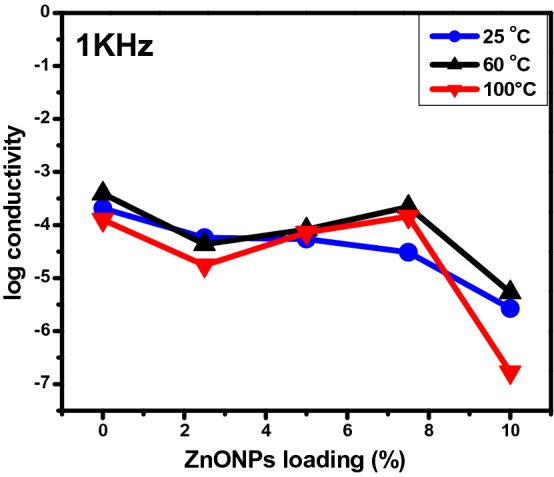
Variation of conductivity of RSNF/ZnONPs nanopaper with ZnONPs loading at different temperatures and 1.0 kHz frequency.

Figure 13 shows the effect of increasing the concentration of ZnONPs on the conductivity at 25, 60, and 100 °C. Measurements were all at a fixed frequency of 1.0 kHz. Only at 25 °C the values of conductivity presented a gradual systematic tendency to decrease. The conductivity of these flexible nanopaper films with added ZnONPs may be varying due to different factors like high water sorption properties, the presence of polar functional groups and free charges, and due to compactness of the films. At room temperature, the conductivity slightly decreased with increased concentration from 2.07 × 10^−4^ to 6.21 × 10^−5^.

Upon increasing ZnONPs to 7.5 wt.%, the conductivity was mostly constant. The same behaviour was noticed at temperatures 60 and 100 °C. With increasing ZnONPs wt.%, agglomerates begin to form leading to the filler particles to being in contact with each other.

Increasing the amounts of ZnONPs enhanced the movement of electrons due to its existing Zn vacancies which modify the bond nature of the ZnO lattice in the neighbouring regions. These Zn vacancies may thus act as trap centres that capture electrons from the conduction band. Activating such trap current carriers leads to a decrease in electrical conductivity. Moreover, the scattering of electrons at domain interface boundaries much decreases the conductivity as an excessive amount of ZnONPs is added.

The noticeable increase in conductivity at 7.5 wt.% ZnONPs can be related to the fact that this amount of ZnONPs could introduce a change only through the chemical nature of ZnONPs, without altering the structure already present in neat RSNF. As for the highest content of ZnONPs (10.0 wt.%), its highly crystalline nature lead to the observed decrease. The other factors like preparation methods, pH of the reaction system, and source of cellulose, were all unvarying**.**

As shown in Fig. 13, the variation of conductivity with wt.% of ZnONPs can is divided into three regions. In region I, at all temperatures the conductivity decreased with increasing ZnONPs, which can be attributed to the moving of charged particles without a continuous conductive path. In region II at 60 and 100 °C the conductivity increase due to a continuous conductive path developed in the RSNF matrix. Increasing temperature provides an increase in free volume and segmental mobility, which permits free charge or electron to hop to another site leading to an increased in conductivity.

#### Dielectric constant (ε′)

The amount of charge that can be stored by a material is reflected by the dielectric property. This amount can be used as evidence that an increase in the free mobile ions or charge carriers increases the conductivity. In addition to the value of electrical conductivity, analysis of the capacitance values also shows the ability of a material to store electric charges. Based on the capacitance value, the dielectric constant of the sample can be determined as in Eq. (6). The dielectric constant (ε′) of all the prepared thin films was measured within the frequency range 50 Hz to 5 MHz, and the temperature range from 25 to 100 °C.

**Figure 14:**
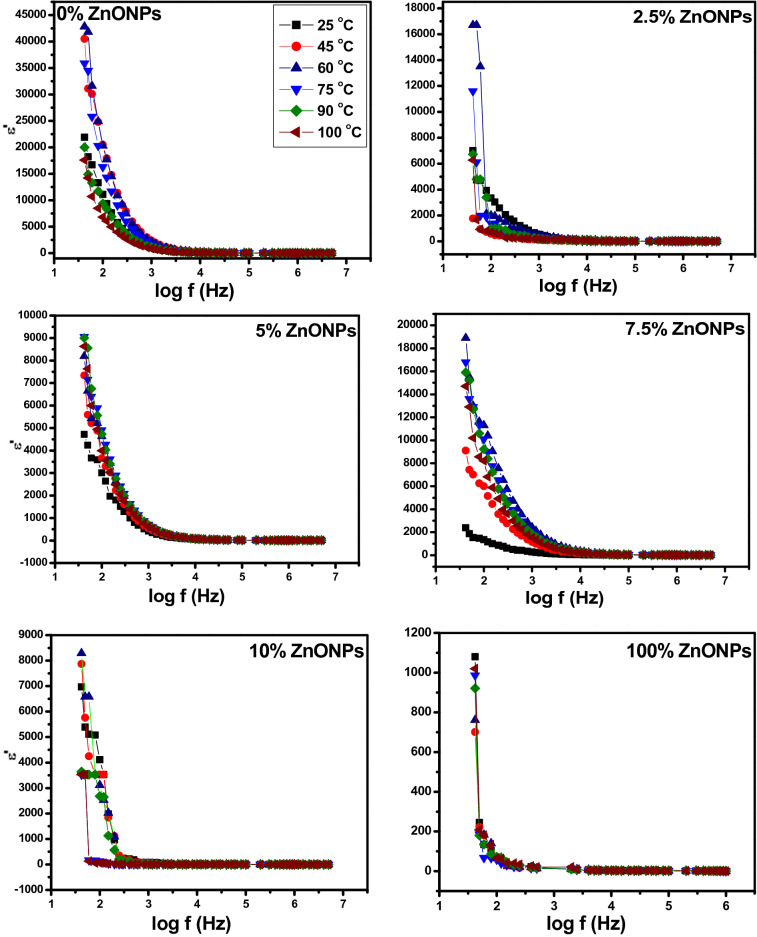
Variation of dielectric constant with frequency at different temperature for ZnONPs and RSNF/ZnONPs nanopaper films.

Figure 14 gives the dielectric constant with the applied frequency at different temperatures for the different RSNF/ZnONPs nanopaper films. It is noticed that the decrease of dielectric constant with increasing the frequency is prominent at low frequency, and takes low value at high frequency. Similar behaviour was obtained for semiconductor materials^10,59^. Dielectric constant ɛ′ measure the polarization of the materials in an applied electric field. The presence of four polarizations (space charge, orientation, electronic, and ionic polarization) are active at low frequencies^60^. All dipoles of polar ZnONPs get aligned in the direction of the applied electric field and thus give rise to a higher value of ɛ′. As the frequency increases, the values of ɛ′ start decreasing, and at certain frequency, it becomes constant because the dipoles cannot follow the alternating field and are unable to align themselves in the direction of the electric field; hence, the values of the ɛ′ decreases. With the frequency going up, the high periodic reversal of the electric field makes it harder for dipoles and charge carriers, which accumulate at the interface of the materials having different conductivity. Hence, the interfacial polarization is decreased which is attributed to Maxwell–Wagner–Sillars (MWS) effect and results in lower ɛ′^61^.

**Figure 15:**
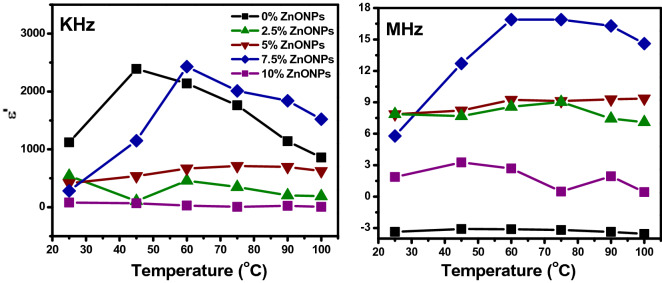
Variation of dielectric constant with temperature at fixed frequency for RSNF/ZnONPs nanopaper films.

Figure 15 shows the variation of dielectric constant with temperature for all films at constant frequencies 10^3^ Hz and 10^6^ Hz. The values of the dielectric constant increase with temperature at a fixed frequency; this is prominent at a low frequency of 7.5 wt.% RSNF/ZnONPs sample. At low temperatures, the dipole is rigid; as the temperature increases the dipoles become free, thus the polarization increases and the dielectric constant increase.

The value of ɛ′ increases with temperature for all films till 60 °C, and then takes constant values. This is due to the greater freedom of movement of the dipole molecular chain.

**Figure 16:**
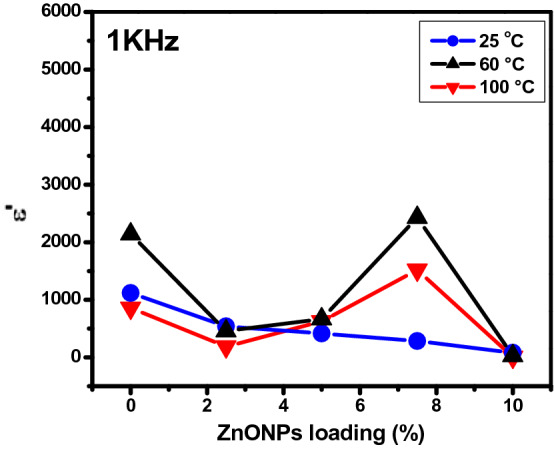
Variation of dielectric constant of RSNF/ZnONPs nanopaper films as a function of ZnONPs loading at different temperatures.

Figure 16 shows the variation of dielectric constant with ZnONPs wt.% at fixed frequency 10^3^ Hz at different temperatures (25, 60 and 100 °C). Increase in the ZnONPs doping leads to a greater number of dipoles that get aligned under the influence of the electric field; hence resulting in greater permittivity and a higher value of ɛ′. At room temperature, the effect is not noticed with increasing the wt.% of ZnONPs. However, at 60 °C the ɛ′ value is 360 with 2.5 wt.% and increase to 2430 with 7.5 wt.% then decrease to 4 with 10 wt.%. This variation is a typical response in the heterogeneous matrix, and is due to the interfacial polarization which occurs at the interfaces of the different constituent phases; hence reducing the dielectric breakdown strength of polymer nanocomposite^62^.

## Conclusions

Low-cost cellulose nanopaper sheets with good mechanical properties and flexibility could be prepared from RSNF and ZnONPs. TEM analysis confirmed the fabrication of spherical ZnONPs with a diameter of 10.5 nm. XPS data show that the interaction between ZnONPs and RSNF is due to formation of RSNF/ZnONPs nanocomposites by electrostatic and hydrogen bond interaction. Uniform dispersion of ZnONPs within the RSNF matrix is confirmed by SEM. Electrical properties of RSNF nanopaper were affected after addition of the ZnONPs. The ac-electrical conductivity increased with increasing frequency. At low frequency, ε′ of the films was enhanced, while at high frequency the ε′ was low in accordance with Maxwell Wagner model. Adding ZnONPs to the RSNF imparted it interesting electrical properties such as high conductivity, i.e. higher semiconducting properties, and dielectric constant originated from the semiconductor properties of the formers. On the other hand, RSNF with its high flexibility and film forming property allowed preparing of flexible semiconductor films with used with ZnONPs in the powder from. Based on the measurement of the electrical properties, the prepared RSNF/ZnONPs nanopaper could find potential applications in electrical devices.

Low activation energy values from dc-measurements explained that the conductivity in the present samples is of electronic nature, in addition to the fact that activation energy values deduced from dc-conductivity measurements were smaller than those deduced from ac-conductivity. Moreover, the exponent *s* values obtained from power law confirmed that the transport is through the ion hopping mechanism. The prepared nanocomposite films showed good homogeneity and acceptable tensile strength properties until 7.5 wt.% of ZnONPs loading. This supports the prepared nanocomposite films to be an excellent choice for various electronic devices applications.

## Data Availability

All data generated or analysed during this study are included in this published article.
